# Medical and Philosophical Intelligence

**Published:** 1816-07

**Authors:** 


### MEDICAL AND PHILOSOPHICAL INTELLIGENCE.

*To the Editors of the London Medical and Physical Journal.*

GENTLEMEN,

THE last number of the Repository furnishes "a Case of Rabies cured by Blood-letting." The disease was in a dog; and it is most sincerely to be wished that the whole history had been related with a perspicuity and minuteness equal to its importance. This remark will be pardoned when it is added, that the account originates with a gentleman, who, though extremely well informed, yet, for want of professional knowledge, could not be aware of the difficulties of his subject. The following is the history in as few words as possible. The remarks will do no injury if circulated to every huntsman and master of hounds through the kingdom—*pauca sed quæ legat ipse venator.* It must be by the combined attention of such with medical men that we are to expect satisfactory evidence.

In December, 1813, hydrophobia had occurred in a human subject, in the neighbourhood of Newcastle. Several dogs were killed, and a gentleman who keeps a pack of hounds, apprehending rabies in his kennel, killed all those dogs which showed marks of having been bitten. Notwithstanding this precaution, in seven or eight months, nine or ten hounds had, in succession of from one to two months, been attacked with rabies. They were separated from the rest, and all died. The last case occurred in September, when, to use the author's words, it was thought that the *disease was eradicated.*

In the following Sept. [1815], shortly after the hounds had been collected from their quarters [the cottagers among whom they are quartered in the summer], rabies again made its appearance. "The dogs quarrelled one night, and two were very severely bitten. Though the owner conceived that the biting might be in some measure accidental (possibly from the rencounter of so great a number, some of them strangers), yet, calling to mind the mortality of the former year, he judged it prudent to have these two destroyed. Not many days elapsed, however, before a bitch which had been *at walk*, and which had hunted two or three times since her arrival, exhibited unequivocal symptoms of rabies; and from this period the disease continued, as in the previous instance, showing itself amongst the dogs, at intervals of three, four, five, or more weeks, until last February. During this latter period of five months, *thirteen* of the pack had the disease; of which number *twelve* (together with three pointers) died, and *one hound recovered.*"

These repeated losses, of course, induced a corresponding degree of diligence and caution. Calomel, James's powders, and blood-letting, were the principal remedies; and, as they seemed to produce produce some alleviation of the symptoms, hopes were entertained of greater success if the remedies should be applied earlier. "An opportunity presented itself in January last. A fine healthy dog, six or seven years old, though apparently well the evening before, was observed early in the morning to have something very peculiar in his manner; and, on trying him with food, he was found desirous of eating, but unable to swallow. On a closer examination, the owner ascertained that he had all the incipient characteristic symptoms of rabies. He had him immediately bled to a much greater extent than any of the others had been; in fact, the bleeding was continued until the animal nearly *fainted,* and *fell down.* Five grains of calomel, and ten of James's powder, were also given.* As he seemed faint, and much reduced by the bleeding, he was not blistered till the evening; when a severe blister, reaching from the fore-part of the throat to the breast, was applied. He appeared much exhausted by the bleeding and the other remedies; but soon regained his appetite and strength, without the use of any more remedies, or the intervention of any untoward symptom. Still it was thought advisable to keep him chiefly on milk diet for a week or ten days, with very little, if any, animal food during that time.

"Two other dogs have since had the disease; but unluckily it had arrived at a more advanced stage before it was detected, and, although similar means were used, they proved ineffectual.

"Some of those that died were dissected. The throat and gullet were found of a *dark red* colour, which, about the cardia became, as the owner very emphatically terms it, of a *gangrenous green.* Their throats, while alive, were all much swelled. The one that recovered appeared to have much less of this fullness of the throat. All of them lapped milk or water very readily: indeed they evinced an eagerness for fluids, and would often continue lapping for the space of half an hour, without being able to get a drop over. This the proprietor thinks arose from inability to accomplish the movement of the jaw necessary to deglutition, for he remarked, that, when irritated, they were able enough to bite."

All this account is good as far as it goes, which is most certainly to an extent that ought not to be overlooked, inasmuch as it furnishes very important facts. But neither the diagnostic symptoms of the disease, nor with the account of the dissection are completely satisfactory. There is nothing in the whole which may not be ascribed to what the huntsmen call the *distemper*, or what is more accurately expressed by the housewife, who, when she finds a mortality among her poultry, concludes that the "ground is tainted." As far as the account informs us,

"* It is proper to observe that the general effect of these remedies was purging, and frequently vomiting. A great quantity of flowers of sulphur, likewise, was used in the hounds' ordinary food, to keep them cool and open; but not on the day before hunting, for fear of cold or wet."

the the hounds, whether individually or in numbers, were seized with the complaint soon after their arrival from "*quarters*." That is, they joined the pack which appeared in health, and entered the kennel in health; but, like sailors who, under similar circumstances, enter a ship, the crew of which appear in health, or recruits who are confined to close quarters, they [the new comers] sickened. Most of them died; and the survivors, as well as the old pack, continuing well, the disease was supposed to be *eradicated,* till a fresh set of hounds, unaccustomed to the atmosphere of the kennel, arrived from their quarters, and sickened also. All this is offered with no view to discourage communications from those who are unaccustomed to medical precision, but, from the recollection of a somewhat similar incident, to recommend the following mode of conducting such inquiries.

1st. Whenever a fresh hound is admitted into the pack, or one that has been at quarters, or a bitch after an absence for lining, let it perform quarantine in the manner enforced at the Menelhunt. By this means you will ascertain whether such new comer brings a disease, or is infected by the kennel.

2d. Whenever a dog is suspected to die of hydrophobia, select two or three to be inoculated with his saliva. This experiment will be much more satisfactory if made on dogs who have not entered the kennel or pack.

3d. Whenever an epizootic of any kind occurs in the pack, let every dog whose death is not determined on be carefully washed in soap and water, and let the healthy be sent to quarters, but not where there is any danger of their being crowded. Either visit them weekly, or require weekly accounts of them from the cottagers or farmers with whom they are quartered. Such of the diseased as are permitted to live may be nursed in a convenient out-house, which must be afterwards carefully cleansed.

4th. As soon as the kennels are emptied, a quantity of water should be turned through them, after which every corner should be carefully washed with diluted salt of tartar, or hot lime, or both. After this, a number of brasiers, proportioned to the size of the kennels, should be filled with coal and wood in the highest degree of ignition, every aperture not necessary for supporting the fire being stopped. The fires should be fed till the kennels are rendered extremely hot; and, before the fuel is burnt out, the remaining apertures should be stopped, and left so for a fortnight, after which the fresh ventilation should be admitted for a month.

5th. Let every dog that dies, or is killed under the disease, be carefully examined by the neighbouring surgeon; and let not only the diseased parts be described, but the healthy, in a general way.

*Hatton-Garden;* I remain, &c.
*June* 12, 1816. J. A.

☞ The writer is aware that the term *distemper*, used in his remarks, is by no means determinate. He has heard it used by a breeder to designate a disease to which puppies are liable at certain periods periods of their growth; and, at Lord Barkley's kennel, he found it used to designate a disease similar, as he conceived, to the hospital fever in the human subject. The latter is, of course, the sense in which he has applied it in the present instance.

To give the above as much publicity as possible, the proprietors have circulated, without any additional expense to their customers, a loose copy with each of the present Numbers, which they request may be sent to the keeper of the nearest pack or considerable kennel.

---

The following histories, though in the human subject, are not, we admit, entirely free from suspicion. We shall anticipate, and as far as we can, remove one objection which will occur to most of our British readers. Both the subjects are described as earnestly praying when they considered their disease as fatal. This may induce a suspicion, that the whole was the effect of religious melancholy. But we should make large allowances for national customs. Though our continental neighbours may not have more religion than ourselves, they have much more devotion. Among the catholics, the dread of dying *unconfessed* is notorious. Among the Calvinistic protestants, the death-bed repentance is well known to be viewed with equal solemnity; and, among all classes on the continent, the forms of the respective churches are attended to with much more minuteness than with us. A well-known instance lately became a subject of general but ill-understood animadversion. The Princess of Wales complained that her daughter, at an advanced age, remained *unconformed*. This was smiled at by many; but every German informs us, that throughout the empire it is considered as a rite not less important than baptism; and, though it is not called a sacrament by the reformed churches, yet no young person, after a certain age, ventures to be introduced into life without having passed through that ceremony. This, we conceive, may account for the incident mentioned in the paper without the suspicion of melancholy or enthusiasm.

*(From our German Correspondent.)*

*Extracts from* Dr. H. A. Goden's *Observations on the New Method of treating Hydrophobia.*—In the course of a month, the author had four opportunities of treating four different cases of hydrophobia and canine madness in their highest degrees. The first was accompanied with all the dreadful symptoms, and the cure perfect. The second was terminated successfully in the first stage. The two others, which proved fatal, the author did not see till within a few hours before their decease. The chief point in the cure of this violent distemper, the author thinks to be, the watching of the disorder whilst it is unfolding, and in its earliest stage.

[Some remarks follow on the specific nature of the poison, which make us regret that our continental brethren are so little acquainted with Mr. Hunter's mode of reasoning on morbid poisons.]

On

On the 31st of September, the author was ordered by the county counsellor of the district, to go as soon as possible to Schmottstis' fen, symptoms of hydrophobia having appeared in a girl. He found the patient of eight years of age, who had always been healthy. It was then the second day of the disorder. The surgeon of the parish, Mr. Rahl, delivered the following general account of what had passed from the beginning till the author's arrival:—Within these few days a certain uneasiness and shyness had been observed in the girl, she had complained of drawing pains in the back and neck, was restless, startled, moaned, groaned, and sighed in her sleep. Last night she had complained of an impediment in swallowing, narrowness and strangling in her throat, to which soon after, spasmodic choaking and singultus of a peculiar sound had succeeded. When offered drink, she was taken with shivering and trembling, even the mention of it was sufficient to produce the sensation of strangulation, choaking, and hiccup. The symptoms had gradually, but quickly, arrived at their present form. The author saw the patient just after a fit of the most dreadful symptoms had gone off. She sat upright on the straw, covered with a ragged shift only, suffering neither beds nor clothes to be on it, tearing all to rags in the rage of the paroxysm. Her looks had somewhat peculiar in them, a wild staring almost brutal timidity, but different from that look generally observed as a symptom of the status nervosus, shewed not the character of elevation but rather depression of the mind. The eyes flashed like those of cats in the dark, combining in their motion and expression a raw wildness. The pulse was small, contracted, hard, and intermittent, the bowels these three days obstructed, continual strangury, having constant inclination to make water, which came away by drops only, and flowed off involuntarily during the paroxysms.

The disorder was worse by fits, she had intermissions and lucid intervals, the first returned several times a day, encreased towards night in frequency and violence, and lasted from a quarter to half an hour, the lucid intervals continued about the same time. She was capable of predicting the paroxysms, which began with shivering, trembling, strangling, and choaking, which soon ended in a hiccup of a peculiar sound, resembling very much the barking and howling of a dog; terrible gnashing of the teeth, *trismus tetanus*, tonic spasms alternating with convulsions and tremor of the whole body. The patient at the same time demeaned herself furiously wild, beat, scratched, and bit about her, and tore beds and clothes to pieces. The urine flowed involuntary, and she exhibited unnatural strength; whilst the tonic and tetanic spasms lost themselves in trembling and convulsive motions, and the hiccup in strangling without that peculiar sound, the paroxysm went off and the interval took place.

A fit was easily brought on by offering her water to drink, the sight of which at once produced the symptoms; but even the taking of dry substances, such as the medicine in powders, during the lucid intervals, brought on singultus and show of a morbid state

 of of the œsophagus, where there seemed a contraction or impediment which hindered the free passage of the medicine, and caused that peculiar uneasiness which produced these motions in the diaphragm and muscles of the œsophagus. No external lesion of a bitten wound could be found on the body, but the great toe of the left foot was much swollen, red, hard, inflamed, and painful to the touch. This inflammatory swelling extended all along the upper part of the left foot. In the left knee-joint was a hard, swelled, and inflamed gland, but nothing of this kind could be discovered in the groins.

The state of the mind was dull and heavy. A religious melancholy and devotedness were striking symptoms as well in this as in other patients of the kind, all of whom showed a particular and constant inclination to pray.

Through the exertions of the police, it was discovered, that this girl had been bitten, by a strange dog, in the great toe of her left foot about two months ago, of which the parents knew nothing, and consequently could not inform the justice of it. A neighbour's wife, who had witnessed the accident, and had tied up the wound with a rag, now deposed this evidence. No prophylaxis was applied, nor could it be found out whether the dog had actually been mad, to whom he belonged, from whence he came, or whither he was gone; so much was certain, that in that place he was not killed. It is likewise to be observed, that the patient spent several nights, previous to the appearance of the disorder, restless; that she kept hollowing and startling in her sleep, and calling the name of the neighbour's dog, which however was quite well. The state of the disorder and its prognosis demanded quick and strong measures. The tonic spasms, the trembling convulsive motions, the suffering of the urinary organs, the strangury, &c. all proved evidently the seat of the contagious inflammatory process to be in the spinal marrow.* The anxiety, singultus, alteration of voice, and the internal oppression, showed that the plexus cœliacus and nervus vagus, likewise participated; but the head and the sensorium being free, again proved that the centre of the nervous system—the brain, did not as yet sympathize in it.

Confiding in his former experience and success, in the cure of hydrophobia, though not in so high a degree, and encouraged by the experience of Dr. Vogelsang on the same method, the author hesitated not to call it here likewise directly into use.

A vein on the arm was opened, and the blood permitted to flow ad lipothymiam, and till nausea, sickness, fainting, and senseless-

* Without entering into any of our correspondent's theories, we cannot help considering strangury among the symptoms which should always be looked for in rabies. If not a necessary concomitant, it is at least among those which may serve to confirm the diagnosis of the disease.—See Mr. Surr's Cases, which make the first article of our twenty-third volume.

ness ness were the consequence. The quantity of blood taken away at this bleeding amounted to, or even exceeded, a pound. The bitten toe was scarified and blistered. As often as the lucid intervals would permit, four to six grains of calomel, with three grains of musk and sugar were given dry. Though the taking of the medicine was very difficult, on account of the rising of the stomach, singultus and strangling produced by each dose, it nevertheless succeeded when the experiment was repeated. The unguent. hydrargyri cinereum, with opium, was strongly rubbed in the neck, throat, and spine, as often as the lucid intervals would permit.

The author staid with the patient till two o'clock in the morning, then gave the surgeon directions closely to watch the patient, to apply by nine o'clock as many leeches to the throat, neck, and spine, as he could bring on, and to promote their bleeding. The patient soon recovered from fainting through bleeding, appeared more quiet, and by no means so much exhausted as might have been expected from so great a loss of blood in so young a subject. But this quietness did not last long, for in about an hour all the symptoms occurred. The quality of the blood drawn from the vein was remarkable, the author never having observed the like in any disorder; it appeared quite dissolved, watery, thin, and liquid, similar to that often found in people that have been sometime under the water, its colour was of a yellowish grey, resembling purulent or ichorous lymph, its smell disagreeable, strong, and quite different from the natural smell of blood.

On the first of October, in the evening, the author saw the patient again. The disorder was so far changed, that the lucid intervals lasted longer and the paroxysms did not return so often, though they were the same in violence. The sight of water and taking of medicine produced the wonted effects. The spasms and convulsions still returned with increased anxiety and restlessness, without any provocation. The patient felt a dread of death, spoke of it, and sought composure in prayer. After this, the paroxysms returned the same as before. Towards noon, the patient had had a lucid interval of several hours; and only towards night, the anxiety had increased and the paroxysms returned; strangury and constipation remained, the pulse seemed a little softer than before, yet still enormously hard, but no longer intermittent; voice and language were as violent as before, but the countenance seemed more composed and placid. Towards noon the leeches had been applied, and the surgeon had succeeded in making ten of them suck; they bled considerably, and still continued bleeding in some places. Ten powders had been taken, but it was impossible to ascertain the quantity of mercury the patient had swallowed on account of the singultus, &c. The bleeding was now repeated, and again five teacups-full of blood taken away without producing fainting. The powders were repeated and the dose of calomel increased to eight grains, the embrocation continued.

On the 3d of October, in the evening, when the author again visited his patient, he found the state of her disorder much altered for for the better. The quiet which had immediately followed the second bleeding had lasted longer, and only towards morning anxiety had returned and ended in convulsions. The paroxysms were, however, not so violent, the spasms no longer tonic, but rather a kind of trembling convulsive motion, the barking, hiccup, and strangling, occurred only when excited by the swallowing of the medicine, as did also the asthmatic oppression on the chest. The intervals were much longer, but no sleep as yet. A particularly favourable change had taken place in the looks of the patient, they had become milder and more placid, one might almost say more human; their former ferociousness was changed into a languor, and the expression of countenance was more passive; the strangury had entirely gone off; a diarrhœa had taken place this afternoon, the patient having had eight purging stools. During and after these symptoms, the case had evidently taken a favourable turn.

The medicine was continued, but she now took but four grains of calomel, with three grains of ammonium carbonicum pyro-oleosum instead of the musk; the embrocation was also continued. There were no signs of salivation. The surgeon was directed to repeat the bleeding, in case the paroxysms should again increase in number or violence, but not else, and to give three grains of calomel regularly every two hours, as soon as the spasms and singultus no longer made the taking of medicine so very troublesome.

October the 4th.—The patient continued mending; had slept a few hours very comfortably for the first time. The paroxysm had but once appeared that day without being excited; swallowing was easier, no singultus; the paroxysms were no longer convulsions, but merely horrors. On the evening, she drank for the first time a cup of coffee, with some difficulty, but with neither spasms nor hiccup; she had four purging stools. The surgeon was directed to give two grains of calomel and two grains of ammonium every two hours, but to reduce the dose of calomel to one grain, should the purging increase or become colliquative. No signs of salivation.

October the 6th.—On visiting the patient, the author found her almost entirely recovered; all the dreadful symptoms, even the dread of death and the melancholy, having left her since the 4th. Her appetite had returned, and she had actually already taken some broth with a biscuit and some wine; her voice and speech had become natural, physiognomy composed, and sleep regular. The author found her sitting on the bed playing with another child; the motions were liquid, yet only three to four in twenty-four hours. The surgeon was advised to persevere in the use of the calomel for three days longer, yet to give only two grains daily with as much of the ammonium divided into two doses, and afterwards a solution of the extractum cinchonæ in peppermint-water. The suppuration of the bitten place was to be kept up a fortnight longer.

November the 4th.—The girl is perfectly recovered, and there is is no vestige of the late disorder. It is to be observed, that the most debilitating method here pursued, did by no means produce that debility, which, in so delicate an age, might have naturally been expected. It is also remarkable, that neither the internal and large doses of calomel, nor the long continued use of the mercurial embrocation, rubbed in the immediate vicinity of the salivary glands, produced even the slightest degree of salivation. This phenomenon may partly be accounted for from the degenerated and poisoned state of the blood, which, by the contagion, has become so heterogeneous to the organism, as to be no longer a supporting but rather a consuming principle of life; and partly from the medicine standing in a different relation to the morbid than to the healthy organism.

The second case which terminated successfully by the same method, was in a woman of forty-six years of age, who had been bitten, with two more, by a mad dog at Langowerk. The author, on being informed of the accident, directed the surgeon, Mr. Pfeifer, to pursue the following prophylactic method:—The bitten place was scarified, cupped, and blistered; application of leeches to the wound and its vicinity, seems to be very appropriate, the author has often tried it, but could never succeed in making them suck, as they could not be brought to bite, but shrunk together and fell off. Might their sucking not be taken as a proof whether the dog had been mad or not? The suppuration of the bitten place was kept up for four weeks. On the ninth day after the accident happened, the patients were bled, according to age and constitution. On the tenth, they took calomel, adults ten grains for a dose; mercurial embrocations were rubbed in the neck, so as to produce salivation. The author makes no use of belladonna, the chief indication appearing to be, to remove the inflammatory disposition and the plethora in the lymphatic and glandular system, where the contagion is seated. It is good, if mercury is taken in such doses as to produce diarrhœa. This method proved effectual in two of the patients, who afterwards showed no farther morbid symptoms. But the third, Anna Austin, forty-six years of age, who had been bitten in her left hand and right calf, and who had been treated in the above-directed manner, from the first day, had been freely bled on account of the plethora of her constitution, and in whom the calomel had produced ten liquid stools. The author was informed by the surgeon on the 10th of October, that she, the day before, had complained of drawing pains in her limbs, of stiffness and frequent shiverings; that she was very dull and heavy, afraid of death, restless, anxious, and her sleep interrupted by frights; that she complained of great anxiety, and presaged her approaching expiration. The author went thither directly, and found the patient in the state above-described. She also complained of frequent shiverings, which terminated in convulsive motions of the hands and body, strangury and ischury, disturbed sleep, heavy dreams and startling, oppression on the chest, dyspnœa and inquietude. She also felt much inclination to pray and to religious devotedness. The skin was parched and hot; the pulse irregular, hard, small, and spasmodic. In the throat, she had a sensation of narrowness, strangulation, difficulty in swallowing, but no particular dread of water, any more than that the taking of all solids and liquids was distressing to her, on account of the narrowness of the swallow and the rising of the stomach and singultus it produced; but the singultus had not that peculiar sound mentioned in the other case. The trembling and convulsive motions were intermittent, and always appeared when her internal anxiety and inquietude increased. Her physiognomy and eyes shewed a little shyness, timidity, and uneasiness; she had neither thirst nor appetite, and no evacuation from the rectum for three days.

The author did not hesitate to declare this case to be the first stage of hydrophobia, and the symptoms those of inflammation in the spinal marrow and the thoracical nerves. The symptoms did not, however, show that high degree of contagion which consists in perfect inflammation of those parts, but without quick aid they would certainly have come to it.

A vein was, therefore, instantly opened on the left arm, and blood taken away, *ad lipothymiam*, till nausea, giddiness, and loss of sense appeared. The blood taken away might amount to twenty or twenty-four ounces, which, though it was not so degenerated as in the other case, yet it appeared dissolved, thin, and watery. Mercury was rubbed in strongly and repeatedly in the neck and spine, every two hours; eight grains of calomel were taken, and two grains of ammonium with magnesia. The suppuration of the bitten wound kept up. This state lasted without any change for two days. On the third, after twenty doses of calomel powders had been taken, the patient had twelve liquid motions. The amendment now went on rapidly, all the symptoms vanishing gradually, and the patient is quite well at the present day. The calomel was continued for eight days longer, but in smaller and not so frequent doses. Here, likewise, the debility was but trifling, and the recovery quick. Strangury and ischury lasted the longest of all the symptoms.

We forbear saying much on the two cases which terminated fatally, observing only that the v. s. was not carried *ad lipothymiam*, and that the calomel given was not by half adequate to the malignity of the contagion, and could, therefore, have no effect.

---

We have received the following from the Secretary of the Royal Medical Society at Edinburgh:—

"What are the chemical changes produced in the air by the growth of plants; and do they, on the whole, purify or deteriorate the atmosphere?"

A set of books, or a medal of five guineas value, shall be given annually to the author of the best dissertation on an experimental subject proposed by the Society; for which all the members, honorary, extraordinary, and ordinary, shall alone be invited as candidates.

The

The dissertations are to be written in English, French, or Latin, and to be delivered to the secretary on or before the 1st of December of the succeeding year to that in which the subjects are proposed. And the adjudication of the prize shall take place in the last week of February following.

To each dissertation shall be prefixed a motto; and this motto is to be written on the outside of a sealed packet, containing the name and address of the author. No dissertation will be received with the author's name affixed; and all dissertations, except the successful one, will be returned, if desired, with the sealed packet unopened.

---

The Bethlem election has finished somewhat different from our expectations. When we heard that the most skilful and experienced physicians were to be selected, we were ill prepared for a hot canvas, and least of all did we expect that men who, according to academic rules, are not yet sufficiently advanced to claim doctorial honours, would have been selected. On this subject we shall endeavour to collect further particulars for a future Number.

The choice of an apothecary was such as must give universal satisfaction. A gentleman, bred to the profession, who had been appointed their steward, conducted himself so well in that office as to claim the patronage of the governors in a trust more immediately connected with his education.

---

*(From our Correspondent in Paris.)*

It is not sufficient to teach the structure and position of the organs—it is also highly necessary to study their uses and their functions. It is physiology which unveils this admirable mechanism, and is, in an eminent degree, *the science of life.*

Two physicians have announced the design of operating a revolution in this interesting branch of the art of healing. The one proposes to re-construct the entire edifice of physiology on new bases, viz. M. Majendie. The other (Coutanceau*) confines himself to preserving the domain of physiology from the invasion of the chemists, who wish to include *the science of life in the science of affinities.* M. Coutanceau examines in turn digestion, hematose,† secretion, nutrition, and assimilation: he seeks to demonstrate by arguments always ingenious, and sometimes incontestible, the vanity of the most generally admitted chemical explanations. Digestion is produced, according to his opinion, in the following manner:—"The alimentary substances, penetrated and acted upon by menstrua, which art cannot imitate, and which derive their essential and characteristic properties from the organic and vital

---

* Revision des Nouvelles doctrines Chemico Physiologiques suivie d'experiences Relatives à la Respiration, par M. Coutanceau. 1 vol. 8vo. Paris, 1814.

† Sanguification, we conceive.

 action action which produces them, experience, in the interior of the digestive tube, a series of alterations which succeed each other in a necessary and determined order, and of which all the chemical phenomena of their nature have not, however, any known relation with those which are the ordinary result of the laws of affinity."

Subjected to the severe tribunal of M. Coutanceau, the beautiful experiments of Lavoisier and Sequin on respiration lose all their eclat. "This doctrine of hematosis, the child of modern chemistry received with so much favour, and which boasts of nearly thirty years experience, is already like an old building, yet standing, but undermined by time, and shaken by the devastations of man, and only waits one more vigorous effort to reduce it to a heap of ruins."

If the reader will turn to our remarks on Lavoisier and Majendie, he will see how little was thought of Lavoisier's chemistry, as connected with physiology, and still less of Majendie's experiments.

---

*Eye-Stones.*—There are few prejudices of the vulgar more prevalent in the United States, than the belief of the efficacy of small calcareous substances, known by the name of eye-stones, to extract foreign particles from the eyes. The origin of these substances was not unknown to naturalists, and the cause of their motions in vinegar was obvious to every attentive observer. The following extract from the personal narrative of Baron Humboldt will confirm the explanation which scientific men must doubtless have given to the movements and efficacy of these mysterious agents.

"Of all the productions on the coasts of Araya, that which the people consider as the most extraordinary, we may even say the most marvellous, is the stone of the eyes, *piedra de los ojos.* This calcareous substance is the subject of every conversation; according to the natural philosophy of the natives, it is both a stone and an animal. It is found in the sand, where it is motionless; but placed singly on a polished surface, for instance on a pewter or earthen plate, it moves when excited by lemon-juice. Placed in the eye, the pretended animal turns on itself, and expels every other foreign substance, that has been accidentally introduced. At the new salt works, and at the village of Maniquarez, the stones of the eyes were offered us by hundreds, and the natives were earnest to show us the experiment of the lemon-juice. They wished to put sand into our eyes, in order that we might ourselves try the efficacy of the remedy. It was easy to see, that these stones are thin and porous opercula, which have formed part of small univalve shells. Their diameter varies from one to four lines. One of their two surfaces is plain, and the other convex. These calcareous opercula effervesce with lemon-juice, and put themselves in motion in proportion as the carbonic acid is disengaged. By the effect of a similar re-action, loaves placed in an oven move sometimes in a horizontal plane; a phenomenon that has given occasion occasion in Europe to the popular prejudice of enchanted ovens. The *piedras de los ojos*, introduced into the eye, act like small pearls, and different round grains employed by the American savages to increase the flowing of tears. These explanations were little to the taste of the inhabitants of Araya. Nature has the appearance of greatness to man in proportion as she is veiled in mystery, and the philosophy of the people rejects every thing that bears a character of simplicity."—*New-Eng. Journ.*

---

*Count Rumford's Legacy.*—Benjamin Count Rumford, an American by birth, whose talents and researches have given him celebrity throughout Europe, lately deceased at his country residence in France. This distinguished philosopher and political economist, mindful of his native country, has bequeathed, with certain restrictions, the whole of his estate to the University, where he had acquired the first rudiments of physical knowledge.* His will provides 1000 cents. a year for the establishment of a professorship, on those departments of Natural Philosophy, which are connected with the improvements of social life. He has also made the University residuary legatee to his whole estate, subject only to certain life annuities.—*New-Eng. Journ.*

---

The supply and the employment of leeches being attended with some difficulties and with some violation of humanity, the following substitute (which forms a species of *cupping)* may, perhaps, be found successful.

A small machine may be made of tin, composed of two chambers, one above the other, joined by a short tube, both chambers being, as far as is convenient, cylindrical. The *lower* chamber may have a wide mouth, with a smooth blunt edge, to apply closely to the flesh; two or three long and narrow apertures being placed perpendicularly in its sides, closed with glass, (but so as to be air-tight,) in order to give a view into the interior. Lastly, the *upper* chamber terminates in a small mouth on its upper side, having a cork stopper. The flesh of the patient being first properly scarified, and then covered with the mouth of the lower chamber of the machine; a hot iron is next to be held within the air of the upper chamber, in order to rarify it, the cork-stopper being removed for this purpose. When this air has been, to a certain degree, rarified, the cork-stopper is to be restored; and the sides of the upper chamber are then to be cooled, that the air which remains may again become condensed. This being accom-

* Count Rumford, then about 16 years of age, attended the lectures of Professor Winthrop in Harvard University in 1770. His early attachment to experimental philosophy is exemplified by the fact, that he constantly walked from Woburn to Cambridge, a distance of nine miles, to attend these lectures.

 plished, plished, the blood may be expected to flow; and whenever the flowing ceases, the operation just mentioned is to be repeated, *toties quoties*, till the due quantity of blood shall have been taken away.—*New-Eng. Journ.*

---

Dr. Clough, 68, Berners'-street, Physician-Accoucheur to the St. Mary-le-bone General Dispensary, and to the Endeavour or Benevolent Society, &c. commences his summer Course of Lectures on the Science and Practice of Midwifery, including the Diseases of Women and Children, on Monday morning, the 7th of July, at half-past ten, and at seven in the evening.

---

*Mr. Salisbury's Botanical Excursions and Calendar of Flora, for June,* 1816.

May 31st.—Battersea Fields and Banks of Thames. The general effect of a late spring is still very evident; but in the meadows there is a luxuriant appearance, and a prospect of a heavy crop of grass.

Ranunculus repens.
Geranium pyrenaicum.
Carex gracilis.
——— riparia.
——— acuta.
Symphetum officinale.
Lychnis Flos Cuculi.
Tragopogon pratense.
Ranunculus sceleratus.
Alopecurus pratensis.
Polygonum Bistorta.
Rhinanthus Crista galli.
Valeriana dioica.
Cratægus Oxycantha.
Equisetum limosum.
Fumaria officinalis.
Sisymbrium amphibium.
Scandix anthriscus.
Narcissus biflorus.
Geranium parviflorum.
Smyrnium olusatrum.
Papaver Argemone.
Scandix Pecten.
Cerastium vulgatum.
Callitriche verna.
Trifolium pratense.

Alopecurus pratensis now in general bloom; but the Cratœgus Oxycantha only partially open, and this in only one place.

June 7th.—Battersea Fields. A wet day, and the excursion deferred till the 11th. The Hawthorn in the hedge in Battersea Fields, where we collected the specimen on the 31st of May, is in great beauty, but the bloom is not fully expanded.

Potamogeton crispum.

———— lucens.

Trifolium pratense.

Senecio Jacobæa.

Carduus palustris.

Orchis Morio.

Poa fluitans.

Alopocurus geniculatus.

Potamogeton perfoliatum.

Bromus sterilis.

Galium Aparine.

Anthemis arvensis.

Sinapis arvensis.

Heracleum sphondylium.

Poa trivialis.

—— pratensis.

Geranium dissectum.

Myriophyllum verticilatum.

Sinapis alba.

Carex vulpina.

Urtica dioica.

Sambucus nigra.

Cochlearia Armoracea.

Trifolium procumbens.

Cerastium viscosum.

Trifolium repens.

Carex depauperata.

Ceratophyllum demersum.

June

June 14th.—Hampstead Heath and Cain Wood.

Gallium pusillum.
Hieracium pilosella.
Veronica officinalis.
Tormentilla erecta.
Hippochæris radicata.
Aira flexuosa.
Plantago coronopus.
Arenaria verna.
Ornithopus perpusillus.
Bromus muralis.
Trifolium striatum.
Carduus lanceolatus.
Ranunculus flammula.
Brium scoparium.
Carex stellulata.
Menyanthes trifoliata.
Eriophorum vaginatum.
Aspidium Felix fæmina.
——— Felix mas.
Stellaria uliginosa.
Veronica Beccabunga.
Cynosurus cristatus.
Carex remota
——— Michaeliana.
Lychnis Dioica.
Bunium bulbocastanum.
Lysimachia nemorum.
Asperula odorata.
Carex muricata
Rhamnus frangula.
Fraxinus excelsior.
Poa angustifolia.
Scrophularia nodosa.
Poa subcærulea.
Geum urbanum.
Carex sylvatica.
Convallaria majalis.
Bryonia alba.
Solanum Dulcamara.

June 21st.—Charlton and Hanging Wood near Woolwich. The Hawthorn is still in bloom, and in great beauty, in many places in the neighbourhood.

Festuca duriuscula.
Hordeum murinum.
Erysimum officinale.
Poa rigida.
Medicago polymorpha.
Ranunculus arvensis.
Lolium perenne.
Alopecurus agrestis.
Lithospernum officinale.
Chœrophyllum temulum.
Ervum hirsutum.
Lithospermum arvense.
Trifolium filiforme.
Lathyrus pratensis.
Briza media.
Chrysanthemum Leucanthemum.
Vicia sativa.
Cynoglossum officinale.
Dactylus glomerata.
Lotus corniculatus.
Salvia pratensis.
Sagina precumbens.
Phleum nodosum.
Urtica urens.
Sherardia arvensis.
Sedum acre.
Ballota alba.
Echium vulgare.
Senecio viscosa.
Raphanus Raphanistrum.
Fumaria claviculata.
Poa compressa.
Bromus racemosus.
Polygonum convolvulus.
Digitalis purpurea.
Galium uliginosum.
Epilobeum rosæum.
Rubus frutriosus.
Cucubulus Behen.
Reseda lutea.
Potentilla argentea.
Vibernum Opulus.
Cornus sanguinea.
Rosa arvensis.
Euonymus europæus.
Crepis biennis.
Malva sylvestris.
Sonchus arvensis.
Vicia Sepium.
Medicago lupulina.
Leontodon hispidum.
Ranunculus arvensis.
Papaver dubium.
Festuca rubra.
Antirrhinum majus.
Saxifraga umbrosa.

*Excursions and Lectures for July.*—At the request of several of the pupils, I have agreed that we meet every Friday in this month, month at the Botanic Garden, Sloane-street, at nine o'clock in the morning.—My work that is in the press, "The Botanist's Companion," will be published on the 10th of this month, and may be had at the Garden.

*Botanic Garden, Sloane-street;* W. SALISBURY.
*June* 28, 1816.

N.B.—The Botanical Lecture will be read, as usual, at the Garden, every Monday, at three o'clock.

---

### REPORT OF DISEASES.

THE town is become considerably more healthy. Inflammatory diseases continue, but with much less violence. Diarrhœas are frequent, but mild; and symptoms of dysentery very rare. Topical bleeding is found extremely serviceable in some cases which continue in a chronic state. Even pulmonary complaints have been much relieved by the free use of the scarifiers and cupping glasses to the skin, over the parts in pain. A case of intus-susceptio proved fatal to a remarkably fine infant only six months old. There was, in this subject, a precocity of strength, and apparently of intellect. Instead of being cleaned like most children, he had, from his fourth month, been regularly in the habit of being put to the stool, and of relieving himself like a much older person. He lived a week without any evacuation downwards, apparently in much pain and constant sickness, though he sucked eagerly to the last. The intus-susceptio was from below upwards. The internal part was nearly mortified, and adhering at the beginning of the duplicature, so that had the child lived, the internal part might have been separated, and ejected with the fæces. Of this an instance occurs in one of our early Numbers, related by Dr. Hull, of Manchester, and noticed with great candour by our valued correspondent Dr. Baillie.

Scarlatina and measles continue very severe, and often fatal. Scarlatina principally by very large gangrenes about the fauces. Small-pox is less frequent and less fatal. The late unfortunate cases have produced a happy effect on the public mind respecting vaccination.

METEOROLOGICAL

### METEOROLOGICAL REGISTER.

*From May the 25th, to June the 26th,* 1816.

Kept by C. BLUNT, Philosophical Instrument Maker, No. 38, Tavistock-Street, Covent-Garden.

| Moon. | Day. | Wind. | Barometrical Pressure. Max. | Min. | Mean. | Temperature. Max. | Min. | Mean. | |
|---|---|---|---|---|---|---|---|---|---|
| | 26 | E | 30·15 | 30· | 30·075 | 57 | 38 | 47·6 | Fair |
| ● | 27 | SE | 30·04 | 30·03 | 30·035 | 56 | 40 | 47·6 | Fair |
| | 28 | SE | 30·12 | 30·1 | 30·11 | 58 | 41 | 39·3 | Fair |
| | 29 | SE | 30·16 | 30·16 | 30·16 | 60 | 42 | 51· | Fair |
| | 30 | SE | 29·89 | 29·89 | 29·89 | 64 | 46 | 54·3 | Fair |
| | 31 | SE | 29·90 | 29·89 | 29·895 | 63 | 43 | 54· | Fair |
| | 1 | E | 30·06 | 30·06 | 30·06 | 61 | 43 | 53·3 | Fair |
| | 2 | E | 30·06 | 30·06 | 30·06 | 60 | 42 | 51·3 | Fair |
| ◐ | 3 | E | 30·05 | 30·05 | 30·05 | 59 | 40 | 50· | Fair |
| | 4 | NE | 30·05 | 30·02 | 30·035 | 57 | 41 | 50·6 | Fair |
| | 5 | N | 29·81 | 29·81 | 29·81 | 56 | 42 | 49·3 | Fair |
| | 6 | NW | 29·90 | 29·88 | 29·89 | 55 | 41 | 48·3 | Rain |
| | 7 | NW | 29·83 | 29·71 | 29·77 | 58 | 42 | 50· | Rain |
| | 8 | NW | 29·60 | 29·54 | 29·57 | 60 | 43 | 51·3 | Rain |
| | 9 | W | 29·70 | 29·70 | 29·70 | 61 | 41 | 51· | Fair |
| ○ | 10 | NW | 29·86 | 29·80 | 29·83 | 61 | 40 | 51· | Fair |
| | 11 | NW | 30·05 | 30· | 30·025 | 62 | 40 | 51·6 | Fair |
| | 12 | NW | 30·16 | 30·16 | 30·16 | 60 | 42 | 51·3 | Fair |
| | 13 | N | 30·12 | 30·07 | 30.045 | 61 | 44 | 52· | Fair |
| | 14 | N | 30·07 | 30·07 | 30·07 | 62 | 41 | 51· | Fair |
| | 15 | NW | 30·06 | 30·06 | 30·06 | 63 | 40 | 50·3 | Fair |
| | 16 | NW | 30·05 | 30·05 | 30·05 | 66 | 42 | 51·3 | Fair |
| ◑ | 17 | NW | 30·04 | 30·04 | 30·04 | 68 | 43 | 53·3 | Fair |
| | 18 | W | 30·02 | 30·01 | 30·015 | 69 | 41 | 53·3 | Fair |
| | 19 | SW | 30·08 | 30·05 | 30·065 | 70 | 40 | 52·6 | Fair |
| | 20 | W | 30·13 | 30·13 | 30·13 | 72 | 40 | 52·6 | Fair |
| | 21 | W | 30·16 | 30·15 | 30·155 | 70 | 42 | 54·6 | Fair |
| | 22 | SW | 30·16 | 30·11 | 30·135 | 71 | 43 | 55·6 | Rain |
| | 23 | S | 30· | 30· | 30· | 68 | 44 | 56· | Rain |
| | 24 | SW | 29·90 | 29·87 | 29·885 | 66 | 42 | 55· | Fair |
| ● | 25 | SE | 30· | 30· | 30· | 65 | 43 | 54·6 | Fair |

RESULTS.

Mean barometrical pressure of the month 30·005
Maximum 30·16, wind at SE
Minimum 29·54, ——— NW

Mean temperature of the month 51·285 deg.

Maximum 72, wind at SW

Minimum 38, ——— E

*Scale exhibiting the prevailing Winds during the Month.*

| N | NE | E | SE | S | SW | W | NW |
|---|---|---|---|---|---|---|---|
| 3 | 1 | 4 | 6 | 1 | 3 | 4 | 9 |

| | Mean barometrical pressure. | Mean temperature. |
|---|---|---|
| From the new moon on the 27th May, to the first quarter, on the 3d June | 30·03 | 50·11 |
| ——— the first quarter on the 3d, to the full moon on the 10th | 29·832 | 50·07 |
| ——— the full moon on the 10th, to the last quarter on the 17th | 30·04 | 51·21 |
| ——— the last quarter on the 17th, to the new moon on the 25th | 30·053 | 54·12 |

