# Erratum

**Published:** 1816-08

**Authors:** 


ERRATUM.

In our last page 74, in middle, for *conformed* read *confirmed.*

